# Clostridial Gas Gangrene ‐ A Rare but Deadly Infection: Case series and Comparison to Other Necrotizing Soft Tissue Infections

**DOI:** 10.1111/os.12804

**Published:** 2020-10-04

**Authors:** Maximilian Leiblein, Nils Wagner, Elisabeth H Adam, Johannes Frank, Ingo Marzi, Christoph Nau

**Affiliations:** ^1^ Department of Trauma, Hand, and Reconstructive Surgery Intensive Care Medicine and Pain Therapy, University Hospital Frankfurt Frankfurt Germany; ^2^ Department of Anesthesiology Intensive Care Medicine and Pain Therapy, University Hospital Frankfurt Frankfurt Germany

**Keywords:** Clostridium, Gas gangrene, Myonecrosis, Necrotizing fasciitis, Soft tissue infection

## Abstract

**Objective:**

Clostridial gas gangrene (GG) or clostridial myonecrosis is a very rare but life‐threatening necrotizing soft tissue infection (NSTI) caused by anaerobic, spore‐forming, and gas‐producing clostridium subspecies. It is the most rapidly spreading and lethal infection in humans, also affecting muscle tissue. The high mortality, of up to 100%, in clostridial GG is mediated by potent bacterial exotoxins. Necrotizing fasciitis (NF) is an important differential diagnosis, most often caused by group A streptococci, primarily not affecting musculature but the subcutaneous tissue and fascia. In the early stages of the infection, it is difficult to distinguish between GG and NF. Therefore, we compare both infection types, identify relevant differences in initial clinical presentation and later course, and present the results of our patients in a retrospective review.

**Methods:**

Patients diagnosed with GG from 2008 to 2018 in our level one trauma center were identified. Their charts were reviewed retrospectively and data analyzed in terms of demographic information, microbiological and histological results, therapeutic course, outcome, and mortality rates. The laboratory risk indicator for NF (LRINEC) score was applied on the first blood work acquired. Results were compared to those of a second group diagnosed with NF.

**Results:**

Five patients with GG and nine patients with NF were included in the present study. Patients with GG had a mortality rate of 80% compared to 0% in patients with NF*.* In eight patients with NF, affected limbs could be salvaged; one NF underwent amputation. LRINEC did not show significant differences between the groups; however, C‐reactive protein was significantly increased (*P* = 0.009) and hemoglobin (Hb) was significantly decreased (*P* = 0.02) in patients with GG. Interleukin‐6 and procalcitonin levels did not show significant difference. Patients with GG were older (70.2 *vs* 50 years). Of the isolated bacteria, 86% were sensitive to the initial calculated antibiotic treatment with ampicillin‐sulbactam or imipenem plus metronidazole plus clindamycin.

**Conclusion:**

Both GG and NF need full‐scale surgical, antibiotic, and intensive care treatment, especially within the first days. Among patients with NSTI, those with clostridial GG have a significantly increased mortality risk due to early septic shock caused by clostridial toxins. In the initial stages, clinical differences are hardly detectable. Immediate surgical debridement is the key to successful therapy for NSTI and needs to be performed as early as possible. However, patients should be treated in a center with an experienced interdisciplinary intensive care team based on a predetermined treatment plan.

## Graphical abstract

Patients with soft tissue infections caused by gas forming clostridia were compared to patients with necrotizing infections caused by other agents and differences were investigated. Patients with clostridial gas gangrene have a significantly increased mortality risk due to early septic shock caused by clostridial toxins. In the initial stages, differences are hardly detectable. Early diagnosis and immediate surgical intervention are the key to successful therapy.

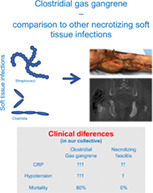

## Introduction

Necrotizing soft tissue infections (NSTI) are characterized by the presence of toxin‐producing bacteria, extensive tissue destruction, and fulminant inflammatory progression, leading to sepsis, multi‐organ failure, and, finally, if untreated, death^1^. Mainly, two types of NSTI are described depending on the microbial agents. Polymicrobial infections are type I infections, while monomicrobial infections are type II infections, which are most often caused by *Streptococcus pyogenes* ^1^, ^2^, ^3^. NSTI can affect any layer of the soft tissue. However, necrotizing fasciitis (NF) is characterized by extensive necrosis of the fascia and the overlaying subcutaneous and skin tissue. Initially, in contrast to clostridial myonecrosis, muscle tissue is not involved. However, in advanced stages, it also affects the musculature^4^, ^5^.

Clostridial gas gangrene (GG) or clostridial myonecrosis is a life‐threatening soft tissue infection caused by anaerobic, spore‐forming clostridium subspecies. It may occur spontaneously, often with the background of abdominal pathology or malignancy, or as a result of a traumatic injury^6^. Clostridial GG has to be differentiated from non‐clostridial GG, a term which is used for any gas‐forming soft tissue infection caused by bacteria other than clostridia^7^.

Historically, GG was observed as a complication of battlefield injuries. During World War I, GG occured in 5% of wounds^6^. In a civilian context, approximately 1000 cases are reported per year in the USA, of which 50% of cases occur after traumatic injuries, 30% postoperatively, and 20% spontaneously, most often associated with malignancy^6^.

Today, trauma is responsible for up to 70% of the cases of GG; other predisposing conditions are bowel and biliary tract surgery, intramuscular injection, retained placenta, and intrauterine fetal death^8^.

Almost 80% of those infections are caused by *Clostridium perfringens*, which usually requires an extensive penetrating trauma. Further pathogens are *Clostridium septicum*, *Clostridium novyi*, *Clostridium histolyticum*, and *Clostridium sordelli*, the latter of which is commonly found in a gynecological context^8^, ^9^, ^10^.

Spontaneous GG is mostly caused by *C. septicum* and occurs frequently in patients with gastrointestinal portals of entry^9^.

Infected patients who do not receive adequate, immediate surgical treatment present mortality rates of up to 100% and death occurs within 2 to 4 days after hospital admission^6^, ^8^, ^11^.

Further factors that increase mortality are advanced age, infection of the trunk, underlying diseases, and shock ^6^, ^12^.

An anaerobic environment is necessary for progression of clostridial infections. Thus, deeply penetrating injuries are more likely to develop an infection than more superficial wounds ^12^. Blood supply is severely impaired by occlusion of vessels caused by toxin‐stimulated platelets, leukocytes, and endothelial cells, which form intravascular aggregates causing thrombosis ^1^.The presence of these aggregates means the ability of leukocytes to cross the endothelium into infected tissue is decreased and hypoxia reduces the function of neutrophils^1^.

The fulminant clinical and histological features of an infection with clostridia are mediated by potent bacterial exotoxins^8^, making clostridial myonecrosis the most rapidly spreading and lethal infection in humans ^13^.

The primary toxin to mediate the effect of *C. perfringens* is alpha‐toxin, a zinc metallophospholipase with phospholipase C and sphingomyelinase activity^14^. Alpha‐toxin is thought to be the major factor for tissue pathology leading to muscle necrosis and hemolysis^6^. Vascular permeability is increased (capillary leak) and myocardial function is reduced, leading to bradycardia and hypotension, and, finally, resulting in shock^15^. The second major toxin is perfringolysin O, or theta‐toxin, a pore‐forming toxin^14^, ^16^. The major toxin of C. septicum is also called as alpha‐toxin and is an aerolysin‐like pore‐forming toxin, secreted in an inactive form, which is oligomerized on the membrane of the host cell. It then forms a pore in the membrane, leading to cell lysis^14^. It produces beta‐toxin (DNAse), hyaluronidase (gamma‐toxin), and oxygen‐like labile hemolysin (delta‐toxin)^9^.

Clinically, NF and clostridial GG present in a similar manner, especially in the early stages of the infection, and it is not easy to draw the difference between the two. However, clostridial GG oftentimes shows an even more dramatic course, with increased mortality. In searching the literature, most often only case reports or series with small numbers of patients can be found^17^, ^18^, ^19^, ^20^, ^21^, ^22^, ^23^, ^24^; to the best of our knowledge, none of them focus on differences between GG and other necrotizing soft tissue infections. As an immediate diagnosis and surgery is the only way to save the patient's life, attention must be focused on clinical symptoms. Therefore, the purpose of this study is, first, to investigate and show differences in clinical presentation, clinical course, and outcome, as well as laboratory markers of patients with clostridial GG (myonecrosis) and other necrotizing soft tissue infections. Second, we present the results of our patients treated for NF and clostridial GG to provide recommendations concerning surgical, antibiotic, and intensive care treatment. Third, we want to draw attention to the importance of early diagnosis of this entity and underline the significance of early and determined surgical debridement, which is essential to save patients' lives.

## Patients and Methods

### *Ethics Approval*

The study was performed at the University Hospital of the Goethe University Frankfurt, with approval from the institutional ethics committee (19–295).

### *Patient Data*

As a first group, all patients diagnosed with clostridial GG over an 11‐year period, between 1 January 2008 and 31 December 2018, in our level one trauma center were identified. As a second group, all patients diagnosed with other necrotizing soft tissue infections (NF, respectively, necrotizing cellulitis) over a 2‐year period, between 1 January 2017 and 31 December 2018 were identified. Patients with NF before 1 January 2017 were already evaluated, with results presented in another article^25^. All patients' charts were reviewed retrospectively.

Diagnosis was based on clinical, microbiological, radiological, and intraoperative findings, as well as on histopathology results.

All patients underwent surgery and microbiological as well as histopathological samples were acquired intraoperatively. Patients were analyzed retrospectively in terms of demographic and social information (gender, age, and comorbidities).

Isolated pathogens and corresponding antibiotic treatment were reviewed. The laboratory risk indicator for NF (LRINEC) was applied on the first acquired blood work^26^.

The way of admission, clinical presentation, and neurological state were analyzed, and diagnostics and the time from admission to operating theater were evaluated. Furthermore, the course of infection and therapy was investigated in terms of anatomical site, etiology, number of days hospitalized, and length of stay in intensive care unit (ICU). The number of surgical interventions and complications were documented.

Medical and socioeconomic outcome in terms of survival, organ and limb salvage, and costs for therapy were analyzed.

The findings in both groups were compared.

### *Index Measure*

#### *Laboratory Risk Indicator for Necrotizing Fasciitis*

The LRINEC is used to differentiate necrotizing soft tissue infections from non‐necrotizing soft tissue infections. It is based on six routine laboratory markers and is calculated as follows: points for C‐reactive protein (CRP) (<15 mg/dL = 0 points, ≤15 mg/dL = 4), white blood cell count (<15/mm^3^ = 0, 15‐25/mm^3^ = 1, ≥25/mm^3^ = 2), hemoglobin (>13.5 g/dL = 0, 11–13.5 g/dL = 1, <11 g/dL = 2), sodium (≥135 mmol/L = 0, <135 mmol/L = 2), creatinine (<1.6 mg/dL = 0, ≥1.6 mg/dL = 2), and glucose (<180 mg/dL = 0, ≥180 mg/dL = 1) are added. The standard score has a maximum of 13 points. A result higher than 8 points categorizes patients as “high risk” for a necrotizing infection^26^, ^27^, ^28^.

#### *Glasgow Coma Scale*

To evaluate the neurological state, the Glasgow coma scale (GCS) was used. The GCS contains three components (motor, verbal, and eye responses), which add up to a score between 3 and 15, with 15 being the best^29^, ^30^. The score is used worldwide in clinical practice and research ^31^.

### *Statistical Analysis*

Data were analyzed with “R” (R 3.5.1 GUI 1.70El Capitan build [7543]). The continuous variables were presented as medians and median absolute deviation (MAD). The categorical variables were presented by count and percentage. The Wilcoxon‐Mann–Whitney *U*‐test was used for comparisons between two groups. Pearson's χ^2^‐test was used to analyze the independence of two variables. A *P*‐value <0.05 was considered statistically significant. The patients' information was anonymized before analysis.

## Results

### *Demographic Data*

Between January 2008 and December 2018 (11 years), five patients were treated for clostridial GG in our clinic. Four of them were male and one female, the median age was 70.2 years (median = 70.2, MAD = 2.64, minimim = 66, maximum = 75).

Between January 2017 and December 2018, we treated nine patients for NF. Of those, seven were male and two female; the median age was 50 years (median = 50, MAD = 14.9, minimum = 28, maximum = 80) (Table 1).

**TABLE 1:** Demographic data, comorbidities, admission, and symptoms

Patient number	Age	Sex	Comorbidities	Immunosupression	Way of admission	Localization	Skin symptoms at admission
Clostridial gas gangrene							
1	70	M	Decubitus sacralis, arterial hypertension, diabetes mellitus II, hip replacement	Diabetes mellitus II	Transferred	Sacral Spine	Emphysema (total back)
2	68	M	Arterial hypertension, diabetes mellitus II, hypokalemia, obesity	Diabetes mellitus II	Transferred	Left leg Abdomen	Livide colored, epidermiolysis
3	72	F	SCLC, hypothyreoidism, arterial hypertension	Chemotherapy, radiation therapy	Transferred	Thigh trunk	Livide colored, epidermiolysis, distinct tumor
4	66	M	Prostate carcinoma (metastasized), COPD, arterial hypertension, chronic heart failure, atrial fibrillation, chronic renal failure, hip replacement, knee replacement, cholecystectomy	Prednisolon, chemotherapy	Self‐initiated	Gluteal Flank	No rubor, regular finding
5	75	M	NSTEMI, coronary heart disease arterial hypertension, asbestosis, atrial fibrillation, diabetes mellitus II	Diabetes melliitus II	Transferred	Left thigh	Rubor, tumor, emphysema
Necrotizing fasciitis							
1	64	M	ACVB, gastric bypass, lumbal spine decompression, cholecystectomy, apendectomy	Multimorbidity	Ambulance	Left groin	Tumor, secretion, fetor
2	51	M	Glaucoma	—	Self‐initiated	Left leg	Tumor
3	50	M	Arterial hypertension	—	Transferred	Thorax, Neck	Tumor, rubor
4	29	M	Intravenous drug abuse	Intravenous drug abuse	Ambulance	Left leg	Tumor, livide colored, smell
5	31	W	Status after sepsis, renal failure	Multimorbidity	Transferred	Thigh	Tumor, surgical wound
6	28	W	Diabetes mellitus I, retinopathia, nephropathia, polyneuropathy, anemia, anorexia, hypothyreoidism, gastritis	Multimorbidity, malnutrition, diabetes mellitus I	Transferred	Thigh	Tumor, rubor, calor
7	34	M	Hodgkin lymphoma IIIa	Chemotherapy	Transferred	Left leg	Tumor, rubor, pain
8	80	M	Prostata‐carcinoma, arterial hypertension, incontinence	Carcinoma	Transferred	Right shoulder	Swelling
9	57	M	Diabetes mellitus II, knee surgery 5 weeks ago Neoplasia of the pancreas with duodeno‐pancreatectomy, splenectomy, cholecystectomy, partial gastrectomy	Diabetes mellitus II	Ambulance	Left leg	Swelling pain, blisters

Patients with clostridial gas gangrene are shown with gray background, patients with necrotizing fasciitis with white background. COPD, chronic obstructive pulmonary disease; NSTEMI, non‐ST segment elevation myocardial infarction; SCLC, small cell lung cancer.

### *Comorbidities*

Among the patients with GG, two had a medical history of malignancies (small cell lung cancer and prostate cancer), and both of them were receiving chemotherapy when the infection occurred. Three patients were suffering from diabetes mellitus type II.

Of the nine patients with NF, one was undergoing chemotherapy for Hodgkin lymphoma, one had prostate cancer, one had neoplasia of the pancreas in their medical history, three patients had multiple comorbidities, and one had a history of intravenous drug abuse. One patient had diabetes mellitus II and two patients had no relevant medical history. For a detailed listing of comorbidities, see Table 1.

### *Location and Etiology*

**Fig. 1:**
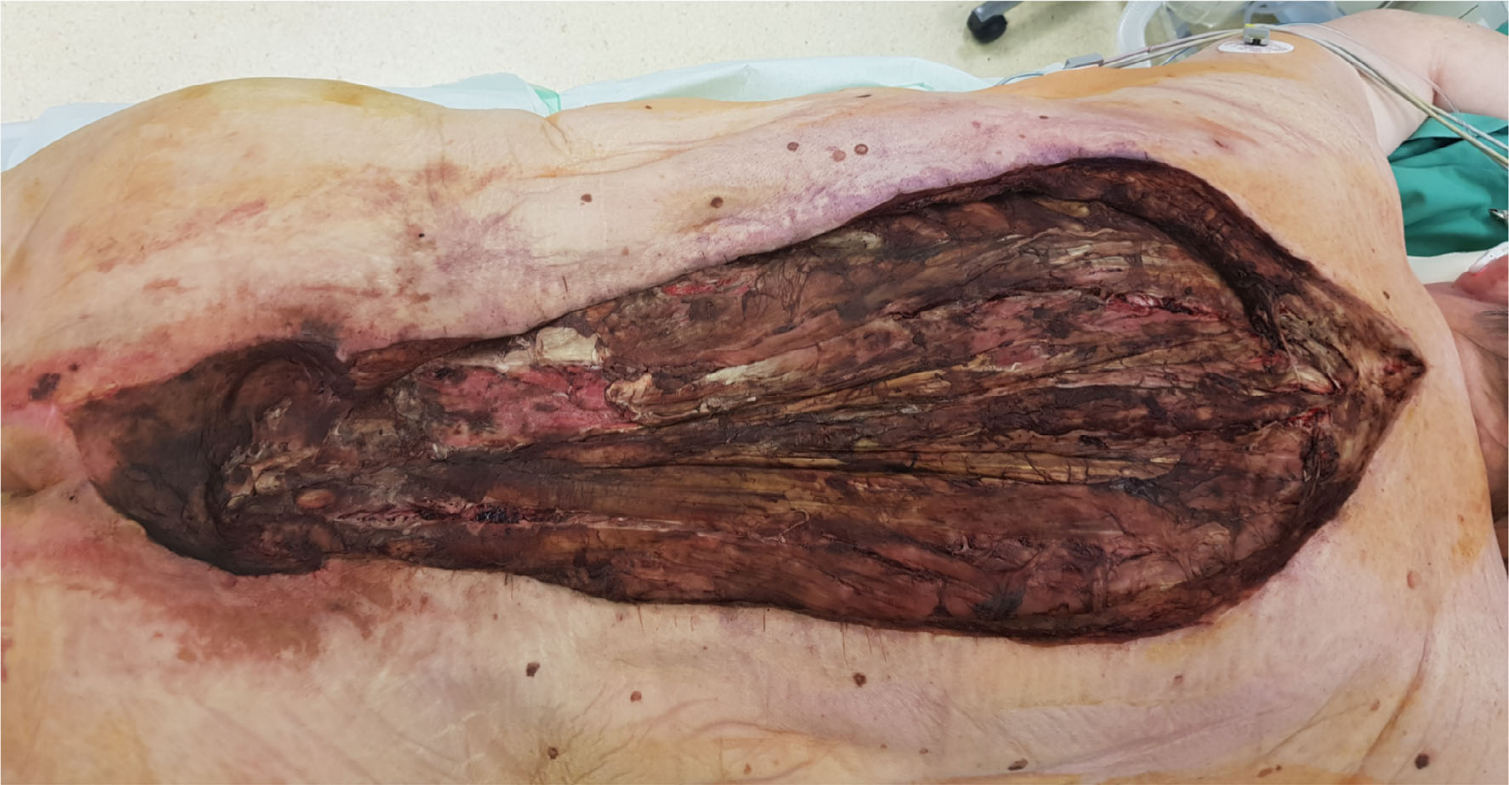
Patient with clostridial gas gangrene caused by *Clostridium perfringens*. Initially the patient had a sacral decubitus. Necrosis of the musculature after extensive debridement of skin and subcutaneous tissue.

Gas gangrene appeared in one patient at the sacrum following a sacral decubitus spreading over the complete back and spine in further course (Fig. 1). In four patients, the infection started in the lower extremities, in one patient subsequently involving the abdomen, in one case the complete trunk, and in one patient spreading over the flank. One infection occurred after an implant removal (femoral osteosynthesis plate), one following a decubitus of the left leg which had developed subsequently to an operation of the spine (dorsal instrumentation), and two spontaneously in patients with malignant comorbidities.

In seven of the nine patients with NF, infection occurred at the lower extremity; in one case the thorax and neck were involved and in another case infection spread from the right shoulder (Table 1). Five infections occurred spontaneously, one developed from an abscess, one after drug injection with parts of a needle remaining in the left leg, one after injection of a glucocorticoid, and one 5 weeks after osteosynthesis of the distal femur.

### *Hospitalization and Costs*

Patients with GG were hospitalized for 10 days (median = 10, MAD = 4, range = 1–14 days) and required intensive care medicine until discharge. None of these patients could be transferred to a regular ward.

Patients with NF spent 32 days in hospital (median = 42, MAD = 9, range = 6–67 days), during which time they were in UCI for 16 days (median = 16, MAD = 4, range = 0–58 days). Only one patient did not require intensive care treatment (Table 2).

**TABLE 2:** Clinical course, treatment, and outcome

Patient number	Time admission to surgery (h)	Number of surgical interventions	Length of stay in ICU (d)	Bacteria	Antibiotic treatment	HBO	Course/complications	Outcome	Time admission to death (d)
Clostridial gas gangrene									
1	1	5	10	*Clostridium perfringens*	Imipenem Clindamycin Metronidazole Vacnomycin Caspofungin	N	Multi‐organ failure	Death	10
2	1.5	2	3	*Clostridium septicum*	Penicillin G Clindamycin Metronidazole Meropenem	N	Multi‐organ failure	Death	3
3	3	1	1	*Clostridium septicum*	Penicillin Clindamycin, Metronidazole	N	Multi‐organ failure	Death	1
4	5	5	14	*Clostridium septicum*	Imipenem Clindamycin Penicillin	Y	Hemorragic shock Multi organ failure	Death	14
5	3.5	4	13	*Clostridium perfringens*	Imipenem Targolid Penicillin Metronidazole	N	Criticall Illnaess PNP	Survived, limb salvage, secondary closure	‐
Necrotizing fasciitis									
1	2	8	4	*Proteus mirabilis* *Morganella morganii* *Bacteroides fragilis* *Candida albicans* *Enterococcus faecium*	Imipenem Clindamycin Teicoplanin	N	None	Survived, limb salvage, mesh	‐
2	72	10	16	*Streptococcus costellatus* *Streptococcus anginosus* *Staphylococcus epidermidis* *Serratia marcescens*	Imipenem Clindamycin Metronidazole	N	Delayed diagnosis	Survived, limb salvage, secondary closure	‐
3	1	9	13	*Streptococcus pyogenes*	Imipenem Clindamycin Metronidazole Teicoplanin	N	Intensive care unit acquired weekness	Survived, secondary closure	‐
4	10	8	20	*Streptococcus mitis* *Eikenella corrodens* *Streptococcus anginousus* *Staphylococcus epidermidis* *Corynebacterium amycolatum*	Imipenem Clindamycin Metronidazole Teicoplanin	N	Acute renal failure Renal replacement therapy Septic shock	Survived, limb salvage, transferred for reconstruction	‐
5	4	6	0	*Acinetobacter baumanii* 4MRGN *Pseudomona aeruginosa*	Daptomycin Ertapenem Colistin Minocyclin Meropenem Cefepim Fosfomycin Fluconazol Ciprofloxacin	N	Acute renal failure Renal replacement therapy	Survived, limb salvage, secondary closure	‐
6	9	3	6	*Staphylococcus epidermidis*	Imipenem Clindamycin Metronidazole	N	Hematoma	Survived, limb salvage, secondary closure	‐
7	4	8	19	*Staphylococcus aureus* *Micrococcus luteus* *Staphylococcus epidermidis*	Imipenem Clindamycin Metronidazole Vancomycin	N	None	Survived, limb salvage, secondary closure	‐
8	9	6	16	*Streptococcus pyogenes*	Imipenem Clindamycin Metronidazole Cotrimoxazole Levofloxacin3	N	Septic shock Acute renal failure ICUAW	Survived, limb salvage, Secondary closure	‐
9	5.5	13	58	*Streptococcus pyogenes* *Candida albicans* *Enterococcus faecium* *Providencia stuartii*	Imipenem Clindamycin Metronidazole Teicolpanin Aciclovir Meropenem Caspofungin	N	Septic shock Multi organ failure Acute renal failure ICUAW HSV‐Pneumonia Prolonged weaning	Survived, above knee amputation, Amputation finger (D2‐4 right hand), Tracheotomy	‐

Patients with clostridial gas gangrene are shown with gray background, patients with necrotizing fasciitis with white background. HBO, hyperbaric oxygen; ICU, intensive care unit.

Of the five patients with GG, four were transferred to our center from other hospitals. One patient's presentation at our emergency department was self‐initiated.

Admisson of patients with NF was self‐initiated in four cases, by ambulance in three of these cases. Five patients were transferred from other hospitals (Table 1).

The costs of therapy during hospitalization, including surgical interventions, hemodialysis, blood products, and intensive care medicine, were €37,792 (median = €37,792.22, MAD = €3567, minimum = €3573.00, maximum = €51,574.22) for patients with GG.

For patients with NF, the costs were €35,178.29 (median = €35,178.29, MAD = €9785, minimum = €4327, maximum = €107,843.71).8

### *Diagnosis*

Preoperatively, clinical examination and laboratory blood analysis were performed in all patients. X‐ray and CT scans were performed in all patients with GG; two of the patients with NF received an MRI instead of a CT scan.

Diagnosis was confirmed surgically by intraoperative findings, as well as microbiological and histopathological results.

**Fig. 2:**
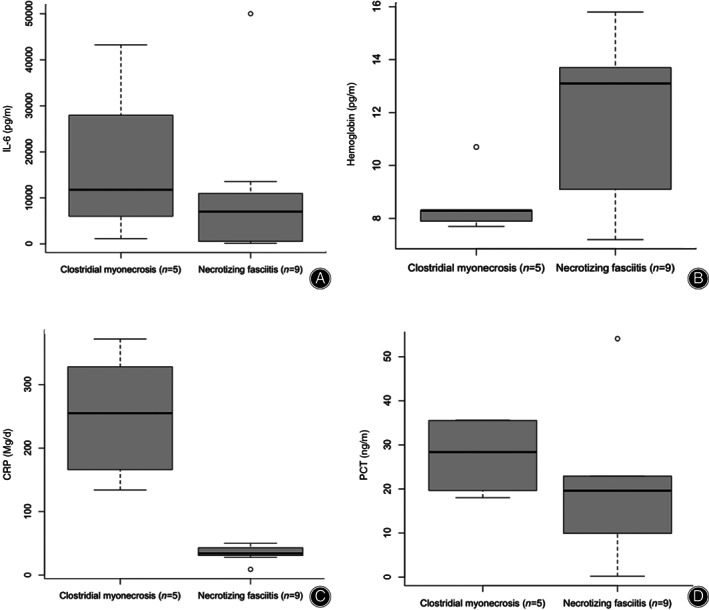
Boxplots of (A) interleukin‐6, (B) hemoglobin, (C) C‐reactive protein (CRP), and (D) procalcitonin (PCT) measured in the first bloodwork acquired. Patients with clostridial gas gangrene showed significantly increased CRP and significantly decreased hemoglobin compared to patients with necrotizing fasciitis (*P* < 0.05).

In laboratory analysis, CRP and white blood cell count were taken routinely; interleukin 6 (IL‐6) was measured in four patients with GG and in seven patients with NF. CRP was highly elevated in all patients and was significantly higher in patients with GG compared to NF (*P* < 0.009) (Fig. 2). IL‐6 was, if acquired, highly elevated in both groups but did not show significant difference between groups (*P* < 0.6) (Fig. 2). There was no correlation between IL‐6‐level and mortality.

The LRINEC was applied retrospectively on the first blood sample taken and a score ≥8 was categorized as high risk^26^. In four patients with GG, the score was ≥8, and in one patient it was 4. Among patients with NF, there were five with a score ≥8 and four had a score <8 (Table 3). There was no significant correlation between the LRINEC and IL‐6 or the LRINEC and PCT levels.

**TABLE 3:** Laboratory results and LRINEC‐score

Patient number	CRP (mg/dL)	WBC (per mm^3^)	Hemoglobin (g/dl)	Serum Sodium (mmol/L)	Serum Creatinine (mg/dL)	Serum Glucose (mg/dL)	(Procalcitonin) (ng/mL)	(IL‐6) (pg/mL)	LRINEC
Clostridial gas gangrene									
1	255	46.66	10.7	138	1.8	102	‐	12,648	**10**
2	372	13.18	7.7	135	1.85	258	35.61	10,853	**9**
3	166	6.7	7.9	132	0.96	206	35.4	1163	**9**
4	134	5.4	8.3	134	1.51	180	18.01	43,259	**4**
5	328	30.11	8.3	136	1.36	228	21.3	‐	**11**
Necrotizing fasciitis									
1	34	20.5	13.5	135	1.19	101	‐	603	**6**
2	40	9.3	13.7	156	1.35	154	9.93	547	**4**
3	34	4.9	15.8	129	2.35	91	54.09	8367	**8**
4	50	8.8	13.1	129	5.17	67	‐	8312	**9**
5	9	11.8	8.2	134	0.52	160	‐	‐	**4**
6	31	13.2	7.2	138	1.62	265	0.22	129	**9**
7	45	0.14	9.1	126	0.91	109	‐	13,549	**8**
8	43	11.2	14.8	138	2.03	178	22.9	5701	**6**
9	28	25.3	10.2	132	1.68	71	19.6	>50,000	**12**

Laboratory risk indicator for necrotizing fasciitis (LRINEC, green columns) applied on the first blood work after admission of patients with clostridial gas gangrene (gray background) and necrotizing fasciitis (white background). In addition, values of procalcitonin and interleukin 6 at admission (IL‐6) are presented. CRP, C‐reactive protein; IL‐6, interleukin‐6; WBC, white blood cell count.

### *Clinical Findings*

At the time of submission, patients with GG all showed hypotonic dysregulation of circulation, three of them already with catecholamine dependency. One patient was intubated when submitted, one was somnolent, and three were neurologically normal with a GCS of 15. Local skin symptoms included emphysema (in two patients), rubor/livide color, tumor, and epidermolysis (in two patients). In one patient, there were no obvious skin symptoms. All patients suffered from strong pain.

Patients with NF tended to be more stable circulatory‐wise. However, in four of the eight patients, a tachycardia (with up to 130 bpm) was documented. Seven patients were awake at the time of submission with a GCS of 15. One patient showed GCS of 15 but was somnolent and in one case a GCS of 4 was documented.

Local symptoms included tumor, swelling, rubor/livide color, calor, secretion, and fetor, as well as pain (Table 1).

### *Microbiology and Histopathology*

In all patients, samples were taken in every surgical intervention and microbiological cultures were applied; the antibiotic regimen was then tailored to the results (Table 1). The microbiological findings of the initial surgical intervention and their antibiograms are listed in Table 4.

**TABLE 4:** Antibiogram of isolated bacteria

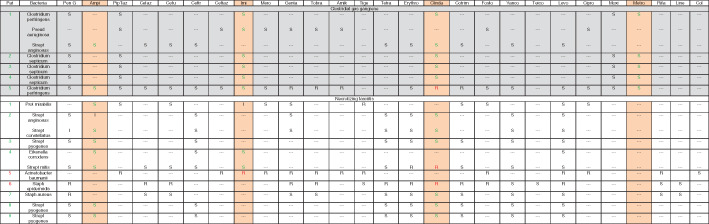

Antibiogram of the bacteria isolated in the first surgical intervention. Rose colored: recommended calculated antibiotic treatment. Patients with clostridial gas gangrene are shown with gray background, and patients with necrotizing fasciitis with white background.

In all patients with GG, clostridia could be identified as a pathogen. In three cases, *C. septicum* was found. Among these, two were spontaneous infections, most likely in the context of a carcinoma. *C. perfringens* was found in the other two patients.

Type I NF was found in six patients, partially in combination with anaerobic agents, and type II NF with *S. pyogenes* (Lancefield group‐A) was found in three patients (Table 2). Histopathological analysis showed extensive necrotizing of the affected fascia in all patients.

### *Therapy and Critical Care Management*

All patients were transferred or self‐initiated presentation to the emergency department of our level one trauma center. Out of the 14 patients included in this study, 13 required intensive care. Immediate treatment after admission included an algorithm‐based therapy according to the recommendations of the Surviving Sepsis Campaign for septic shock^32^.

Patients with GG underwent four surgical interventions (median = 4, MAD = 1, minimum = 1, maximum = 5); the median time from admission to operating theater was 3 h, with the longest time from admission to operating theater being 5 h (median = 3, MAD = 2, minimum = 1, maximum = 5). One patient was intermittently transferred to another hospital for hyperbaric oxygen therapy (*HBOT*), where he underwent three cycles of *HBOT*. The other four patients were not transportable due to, for instance, dialysis and high‐dosage catecholamine therapy.

Patients with NF had eight surgical interventions (median = 8, MAD = 2, minimum = 3, maximum = 13); the median time from admission to the first surgery was 5.5 h (median = 5.5, MAD = 13.1, minimum = 1, maximum = 72). None of the patients with NF had *HBOT*.

Antibiotic treatment was most often started with imipenem, clindamycin, and metronidazole (*n* = 11), and then adapted to the results of the antibiograms. For detailed information on antibiotic treatment, see Tables 2 and 4. Additional supportive care, such as nutritional support and high dose therapy with Vitamin C (6 g per day), was carried out in all patients.

**Fig. 3:**
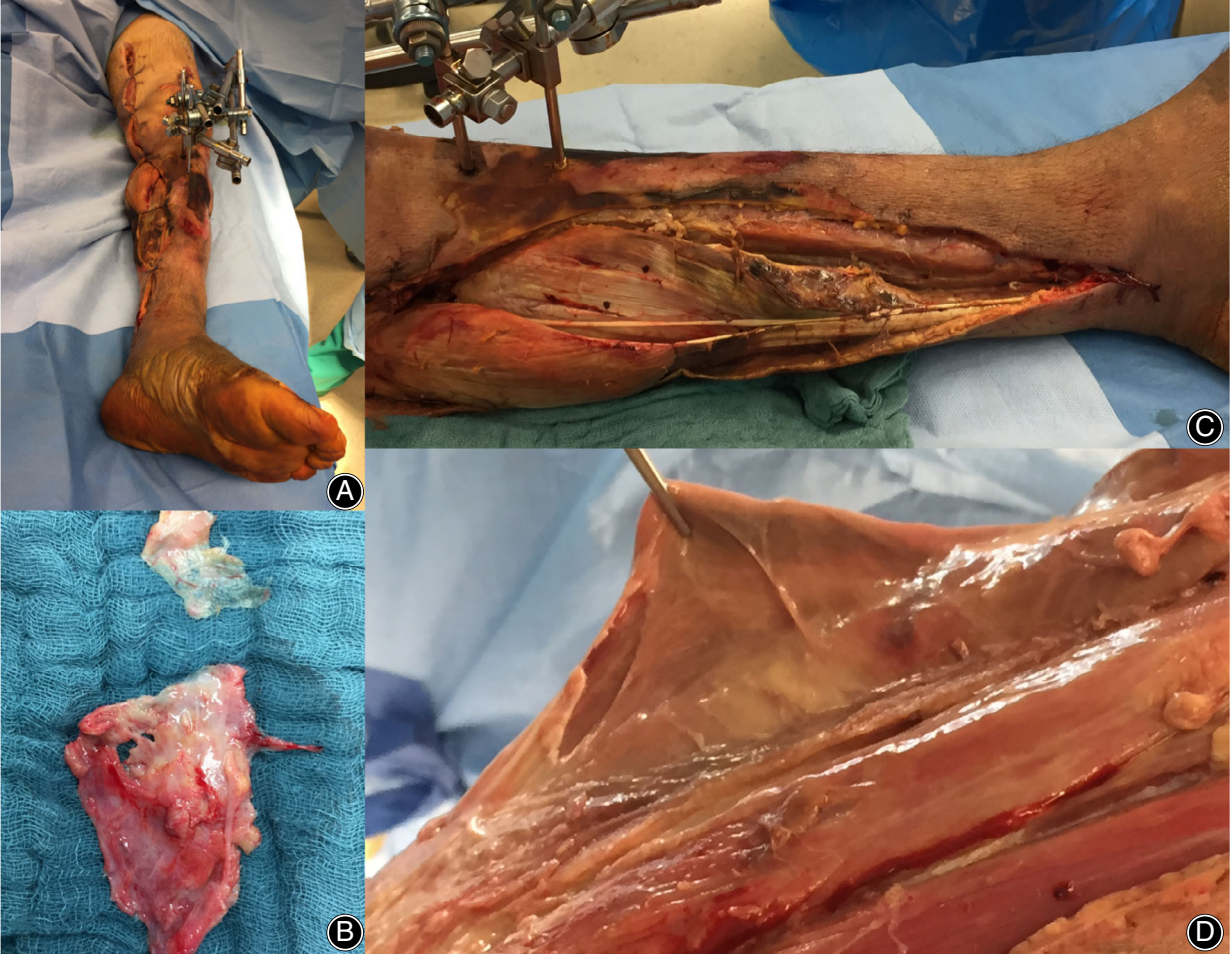
Intraoperative pictures of a patient with necrotizing fasciitis caused by *Streptococcus pyogenes*. External fixator on the left knee after femur fracture and osteosynthesis (A), extensive debridement of the fascia (B), and grayish, disintegrating, chewing gum‐like affected fascia (C, D).

**Fig. 4:**
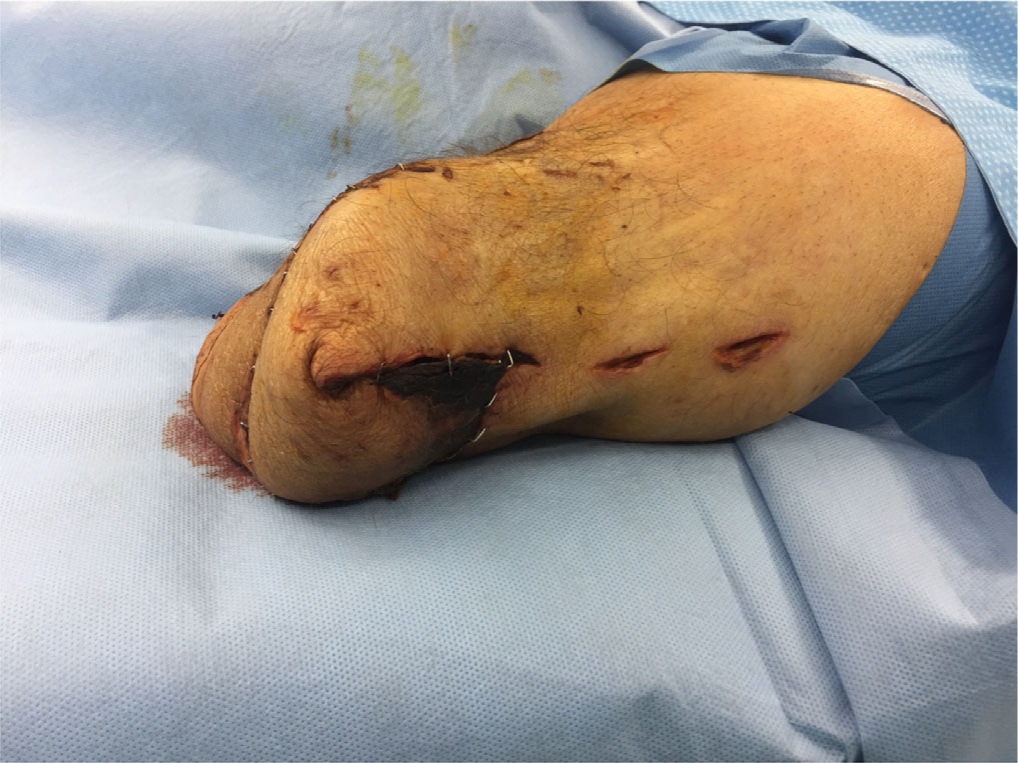
Above knee amputation of the patient in Fig. 3 after multiple debridements.

Surgical treatment included multiple and extensive debridement and, in further course, vacuum sealing, temporary wound closure with, for example, polyurethane foam (Syspur‐derm, Hartmann), and wound closure with skin transplantation (mesh‐grafting), secondary wound closure, or amputation (Figs 3 and 4).

### *Mortality, Outcome, and Complications*

Of the five patients with GG, four died (mortality 80%) of multi‐organ failure. One of these patients developed hemorrhagic shock after surgical intervention. The mean time from admission to death was 6.5 days (median = 6.5, MAD = 4.5, minimum = 1, maximum = 14). One patient survived (20%), was tracheotomized, and developed critical illness polyneuropathy; however, the affected limb could be salvaged and the patient was transferred into rehabilitation after 13 days of intensive care treatment.

All of the patients with NF survived the infection (mortality 0%). Three patients developed ICU‐acquired weakness; four showed acute renal failure, three of whom required renal replacement therapy. In one case, the diagnosis was delayed and time to surgery was 72 h. However, only one patient underwent amputation and affected limbs could be salvaged in eight patients. In one patient, wounds were closed with mesh‐grafting, in six patients secondary wound closure was possible, and one patient was transferred to another hospital for plastic reconstruction (Table 2).

## Discussion

Necrotizing soft tissue infections are a rare clinical entity with a global incidence of approximately 0.4/100,000 per year^33^. Most physicians will only see one case throughout their career^34^, which might provide a reason for delayed diagnosis and inappropriate treatment. The treatment of patients with NSTI is associated with high costs for the healthcare system due to multiple operations, long hospital stays, and extensive intensive care treatment (median = €35,681/patient in our collective).

We have already published data on patients treated for NF in our hospital between 2014 and 2016^25^. In the present study, we share data on patients treated for clostridial GG in the past 11 years and identify differences to patients with NF (treated between January 2017 and December 2018) concerning outcome, mortality, clinical presentation, and treatment.

### *Diagnostics*

Clinical signs of NSTI are dependent on the depth of infection, the anatomical region, and the responsible pathogen^34^. Depending on the stage of infection at the time of presentation, symptoms might be less or more pronounced.

Local signs include swelling, erythema, induration, and pain out of proportion exceeding the margins of apparent skin infection. In further progress, “hard signs” develop, such as bullae and skin ecchymosis, which precede skin necrosis, gas in the tissue with crepitus, and skin anesthesia^34^, ^35^, ^36^. Systemic signs such as fever, tachycardia, confusion, and hypotension, as well as septic shock can be found^9^, ^37^. However, these signs are not specific and clinical diagnosis remains a challenge due to several pitfalls: fever might be missing, cutaneous manifestations can be absent, pain might be attributed to an injury or procedure, and systemic manifestation might be attributed to other causes ^8^.

All of our patients with GG showed hypotension; 60% required catecholamines at the time of presentation. Only 45% of the patients with NF showed hypotension.

**Fig. 5:**
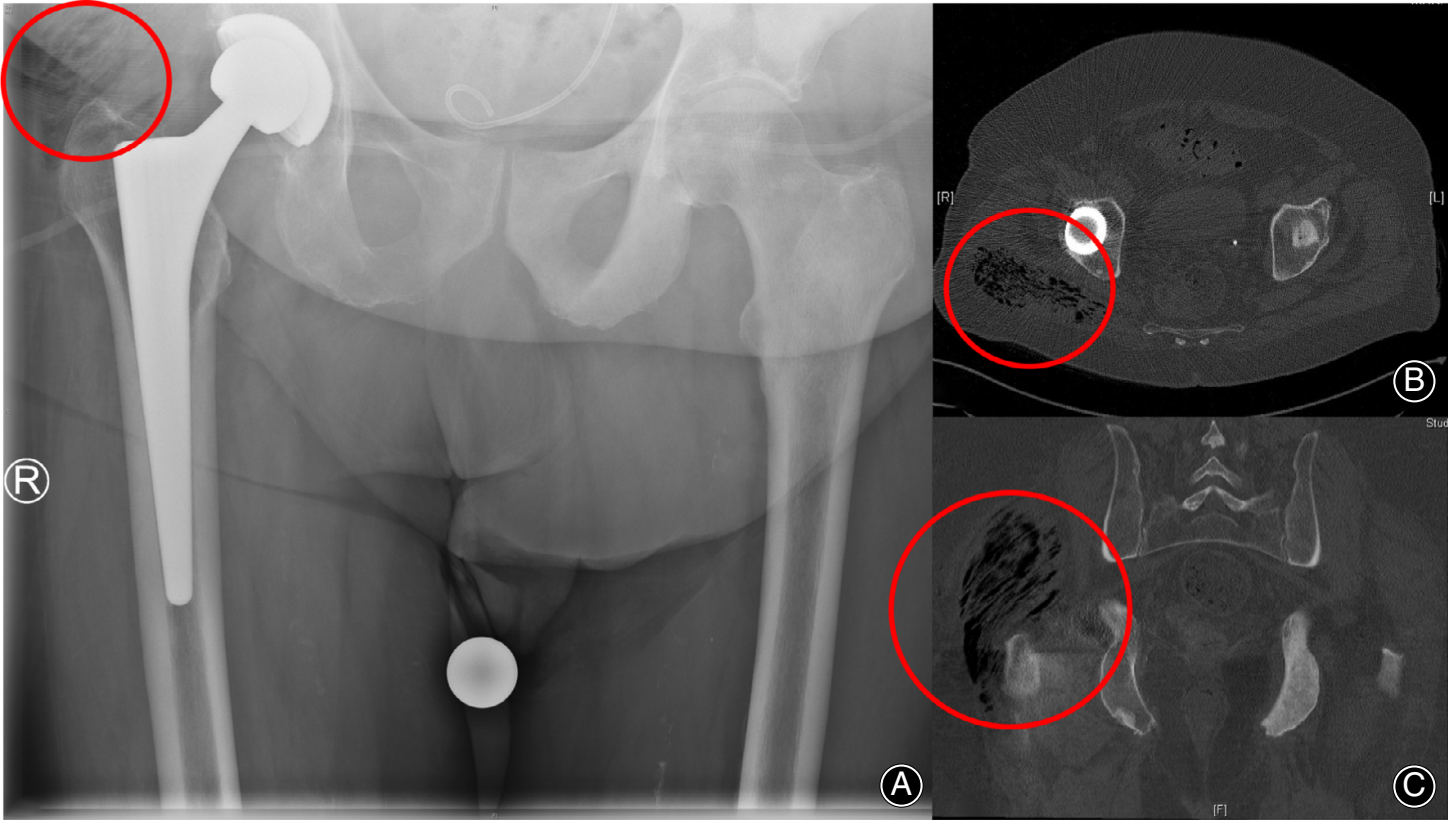
X‐ray of the pelvis of a patient with clostridial gas gangrene caused by *Clostridium septicum* complaining about pain in his right hip. The red circle marks gas in the musculus gluteus (A). CT scan shows the extent of gas formation (red circle) (B, C).

Ultrasound, conventional X‐ray, CT, or MRI can be used for radiological imaging. Ultrasound is immediately applicable but highly dependent on the skills of the examiner. X‐ray might already show gas in the affected tissue in cases of gas‐forming anaerobic bacteria (Fig. 5). CT scans show involvement of the fascia (lack of enhancement after administration of contrast medium), fascial plane thickening, and intramuscular fluid collection^38^. In addition, they provide information about the proliferation of the infection (Fig. 5).

MRI provides high‐definition images of soft‐tissue^8^; however, it must not delay surgical intervention and might not be possible due to septic shock.

The LRINEC helps to further distinguish between non‐necrotizing and necrotizing infections^26^. A cut‐off of ≥6 points has the highest predictive value and a score of ≥8 represents high risk^26^. In the initial blood work of our patients with NF, a score ≥8 was reached in 55% and ≥6 in 78%. Among patients with GG, 80% had a score ≥8 (and ≥6) (Table 3). In literature, the sensitivity of the LRINEC for negative prediction is reported to be 86%‐96% and 57%‐92% for positive prediction^8^.

In addition to C‐reactive protein and leukocytes, IL‐6 and procalcitonin (PCT) were measured (Table 3). IL‐6 is a sepsis‐associated cytokine with high sensitivity for bacterial infection, arising prior to PCT and CRP; however, PCT has been suggested to have the greatest predictive value among the three for bacterial infection^39^, ^40^ and is proposed to be useful in predicting NF or amputation rate ^41^.

Hansen *et al*. found IL‐6 to be associated with severity of infection, amputation rate, and mortality, but it had no significant association to the results of LRINEC score ^42^. Our data neither showed significant correlation between results of the LRINEC and IL‐6 or PCT, nor did we find a correlation between IL‐6‐level and mortality.

Further possible diagnostics, such as the “bedside finger test” or biopsy are described. If NSTI is suspected, diagnosis has, if in doubt, to be confirmed surgically and, subsequently, by microbiological and histopathological findings.

### *Importance of Early Surgical Treatment*

Radical, determined, and early surgical treatment is the most important part of therapy. It is key for successful treatment of NSTI and surgery should not be delayed by diagnostics.

It is reported in the literature that delay of surgical intervention and inadequate debridement might cause significantly higher mortality^3^, ^43^. A delay of more than 12 h can be fatal^44^. However, Latifi *et al*. found time to surgery to be an independent predictor for length of hospital stay but not mortality ^45^.

In a literature review, Ingraham *et al*. reported that expedited interhospital transferal to a specialized center prior to initial debridement is not an independent risk factor for increased mortality or morbidity, even though transferred patients had longer stays on ICU^46^.

The latter observation could not be confirmed in our collective. In the NF group, the median length of stay in ICU was 16 days when presention was self‐initiated and 13 days when patients were transferred from other hospitals. In the GG group, transferred patients spent 6.5 days (median) in ICU when transferred from other hospitals; one patient who self‐initiated presention spent 14 days in ICU. This has to be put into perspective considering that 80% of the patients in the GG group died in ICU.

Even though an interhospital transfer might prolong time to surgery, time from presentation in our hospital to surgery was shorter in patients transferred from other hospitals (GG: median_trans_ = 2.25 h *vs* self‐initiated = 5 h; NF: median_trans_ = 4 h *vs* median_self_ = 9.5 h). As an explanation for this, an increased awareness and, in some cases, the already established diagnosis must be considered.

In both cases (NF and GG), surgical treatment itself should aim to resect all affected tissue; necrotic areas of skin and soft tissue caused by inflammatory thrombosis must be resected^25^, ^47^. Often, multiple debridements are required, even within the first few hours^37^.

Samples for microbiological and histopathological analysis have to be taken during every revision to confirm the diagnosis and adapt antibiotic therapy.

Because of unknown pathogens and suspected anaerobic bacteria, the wound must not be closed until microbiological results have excluded anaerobic agents. An air‐free closed environment, such as that caused by continuous vacuum therapy, can exacerbate anaerobic infection^35^, ^48^. In further course, vacuum therapy helps to condition the wounds and prolong intervals between revisions^35^.

### *Antibiotic Treatment*

For the treatment of necrotizing soft tissue infections, the literature recommends a combination of a broad‐spectrum synergistic penicillin, such as piperazillin‐tazobac or ampicillin‐sulbactam, in combination with clindamycin or a carbapenem. Vancomycin could be added to cover a possibly community‐acquired MRSA^49^. Local incidence of MRSA and respective antibiotic susceptibility should be considered, and gram‐negative, gram‐positive, and anaerobic bacteria should be covered by calculated antibiosis^34^, ^50^.

Guidelines in the USA recommend vancomycin or linezolid plus piperazillin‐tazobac or carbapenem or ceftriaxone‐metronidazole^8^.

The German guidelines, updated 2018, recommend an acyl‐aminopenicillin plus beta‐lactamase‐inhibitor (e.g. piperazillin‐tazobac) or, alternatively, or carbapenem in combination with clindamycin or linezolid (recommended in cases of suspected MRSA).

Alternatively, cephalosporin (group 3) plus metronidazole is used.

The administration of vancomycin primarily is not recommended in Germany, as only 1%–3% of all soft tissue infections are caused by MRSA (*vs* 60% in the USA). If clostridia‐associated gangrene is suspected, vancomycin plus piperacillin‐tazobac or ampicillin‐sulbactam or carbapenem is recommended. Antibiotic therapy should be continued for 7–10 days after surgical consolidation (https://www.awmf.org/uploads/tx_szleitlinien/082-006l_S2k_Parenterale_Antibiotika_2019-08.pdf).

Clindamycin is strongly recommended as it inhibits the synthesis of clostridial exotoxins and lessens their systemic effect^12^. In addition, streptococcal toxin production is inhibited (M‐protein and exotoxin)^35^, ^51^, ^52^. Because clindamycin is bacteriostatic not bactericide, it should always be used in combination^12^.

In our clinic, empiric therapy comprises a combination of ampicillin‐sulbactam/imipenem plus metronidazole plus clindamycin. Reviewing the microbiological results of our patients taken at the first debridement, it is evident that 86% of the isolated bacteria would have been covered. In one patient, *Acinetobacter baumanii* was isolated, which is only sensitive to colistin, while in a second patient, *Staphylococcus epidermidis* was found, which is sensitive to, for example, vancomycin (Table 4).

Because of micro‐thrombosis causing hypoxia and ischemia, local availability of antibiotics in the affected tissue is limited and sufficient local antibiotic concentration is difficult to achieve^33^. Different approaches for local antibiotic delivery have been carried out (e.g. antibiotic‐loaded vitamin D‐granules)^53^. We recommend the local fixation of antibiotics by fibrin spray, as described by Janko *et al*.^54^.

### *Critical Care Management*

As soon as the diagnosis of NSTI is suspected, general resuscitative measures should be performed for management of sepsis and septic shock^32^. Aggressive source control, including surgical debridement, goal directed resuscitation, as well as broad spectrum antibiotic therapy, as mentioned above, are paramount. In addition, adequate fluid administration is essential to restore intravascular volume, maintain adequate end‐organ perfusion and tissue oxygenation, and limit the adverse effects of end‐organ failure. Hemodynamic monitoring may be indicated in some patients with mean arterial pressure <65 mm Hg. To neutralize streptococcal and clostridial exotoxins, intravenous immunoglobulin has been advocated by some; however, definitive data is lacking^55^, ^56^.

### *Difference between Clostridial Gas Gangrene (Myonecrosis) and Necrotizing Fasciitis*

Based on clinical findings, it is not possible to distinguish between clostridial and other necrotizing soft tissue infections. However, a few observations might provide a hint in one direction.

Analyzing the medical history of our patients, we found malignant comorbidities in two of the three patients with spontaneous *C. septicum* infection, an observation in line with the published literature. Kornbluth *et al*. found 81% of *C. septicum* infections associated with malignancy^57^. Way of entry is understood as a defect in the mucosa of the bowel, caused by, for instance, tumor, radiation, chemotherapy, and surgery^9^. *C. septicum* is more aerotolerant compared to *C. perfringens* and, thus, is more capable of initiating infection in the absence of obvious tissue damage.

*Clostridium perfringens* usually requires a deep wound: in our patients, an extensive decubitus subsequent to an implant removal from the femur. Immunosuppression, such as due to diabetes mellitus, helps the progress of the infection, which is fulminant and leads to death, most often within 24 h ^9^, ^58^.

However, immunosuppression was also registered as a promoting factor in seven of the nine patients with NF.

Patients in the GG group were older than patients in the NF group (70.2 *vs* 50 years old) (Table 5). This observation was not confirmed by the literature. Goh *et al*. found a medium age of 55 years in patients with NF^59^, while patients with *C. septicum* infection were 62.5 years old in a review by Srivastava *et al*.; however, patients with other clostridial infections were not included here^9^.

**TABLE 5:** Differences between patients with clostridial gas gangrene and necrotizing fasciitis

	GG	NF
Age (years)	70	50
Gender		
Male	4	7
Female	1	2
Median LOS on ICU (days)	10	16
Median No of operations	4	8
Mortality (%)	80	0
LRINEC	9	8
Hb (g/dl)	8.3 (↓)	13.1 (−)
CRP (mg/dl)	255 (↑ ↑ ↑)	34 (↑↑)
Hypotension	↑ ↑ ↑	↑
Gas in tissue / Emphysema	↑↑	‐
Pain out of proportion	↑ ↑ ↑	↑ ↑ ↑

CRP, C‐reactive protein; Hb, hemoglobin; ICU, intensive care unit; LOS, length of stay; LRINEC, Laboratory risk indicator for necrotizing fasciitis.

Clinically, patients with GG presented in a more severe condition. All of these patients were experiencing hypotonic dysregulation and septic shock. Srivastava *et al*. found hypotension (<100/60 mm Hg) to be the symptom mos tfrequently present at the time of admission (84%), followed by crepitus, erythema, and swelling^9^. Some of the patients with NF were tachycardic but did not show circulatory dysregulation at the time of admission.

Locally, rubor, swelling and pain were the most common findings; in two of the GG‐patients, emphysema was found, which provides an indication for the presence of gas‐forming bacteria (Table 1). Gas in the soft tissue can further be confirmed radiologically with X‐rays or CT scans and provides a hint as to the causing agent.

Comparing the first blood work acquired, we found significantly higher CRP (*P* = 0.009) and significantly lower hemoglobin (*P* = 0.02) in patients with GG. However, LRINEC scores and IL‐6 and PCT levels did not show significant differences between the two groups (Fig. 3).

Further differences became obvious in comparing the number of surgical interventions and length of stay in ICU between both groups. This, again, has to be put in perspective, as patients with GG had a higher mortality rate, so that multiple debridements and reconstructive interventions did not have to be performed.

With regard to the outcome, patients with GG had a significantly higher mortality rate than patients with NF (*P* = 0.01) (Table 5). Generally, morbidity and mortality increased depending on the affected site, being higher if the trunk was involved^12^, which was the case in all four GG patients that died. In the one survivor, only the left thigh was affected. Clostridial alpha‐toxin might be considered as a further reason for the higher mortality of GG patients. Among other effects, it causes intravascular hemolysis and suppresses erythropoiesis, leading to severe anemia. Furthermore, it promotes the release of inflammatory cytokines (e.g. TNF‐alpha, IL‐1, and IL‐6), contributing to toxic shock with hypotension, hypoxia, and low cardiac output^14^. However, in invasive streptococcal infections, exotoxins (A, B, C, streptococcal superantigen) have a similar effect, possibly resulting in streptococcal toxic shock syndrome^35^.

### *Hyperbaric Oxygen Therapy*

The *Tenth European Consensus Conference on Hyperbaric Medicine* recommends the use of *HBOT* in patients with anaerobic and mixed bacterial infections with type I recommendation (strong recommendation) and level C evidence (low level of evidence)^60^.

Clinically, the use of *HBOT* remains highly controversial in the literature. On the one hand, in a retrospective study with 341 patients with NF, the authors showed a significant reduction in mortality and the number of surgical debridement when therapy was supported by hyperbaric oxygen^61^. Other authors recommend the use of *HBOT* in NSTI^11^, ^62^. However, in a literature review on the efficacy of *HBOT* in necrotizing soft tissue infections, Faunø *et al*. found “poor and biased” evidence and the need for randomized controlled trials^63^. The same conclusion was drawn by Anheuser *et al*., who carried out a retrospective multicenter analysis of the influence of HBO on Fournier's gangrene^64^.

The benefit in terms of mortality was not found to be significant by other authors^6^, ^34^, ^65^, ^66^. Yamamoto *et al*. even suspected *HBOT* to be a trigger of metronidazole‐induced encephalopathy in a patient with mandibular osteomyelitis^67^.

Shaw *et al*. recommended the use of *HBOT*; however, in their study, only centers that had a hyperbaric oxygen chamber available were considered.

As there is a “significant limitation on care delivery (p. 352)” in oxygen chambers^34^ and therapy is only available in special institutions, patients have to be stable enough for transport and treatment. Of our patients suffering from GG, only one was stable enough for transport to a center with a hyperbaric oxygen chamber.

Considering the ambiguous discussion in the literature, transportation of a critically ill patient and intensive care for several hours in a hyperbaric chamber can be both challenging and dangerous, and, thus, the risks may outweigh the potential benefits^68^.

### *Limitations of This Study*

Low sample size and the design as a retrospective analysis must be identified as limitations of this study. Iin screening the literature, multiple reports of single cases can be found^17^, ^18^, ^19^, ^20^, ^21^, ^22^, ^23^, ^24^, whereas larger case series in the post‐war period are scarce; Chen *et al*. report on five patients and McGuinness *et al*. on approximately 10 patients^69^, ^70^. Considering that most physicians only see one patient with NSTI throughout their whole career^34^, the sample size has to be put in perspective.

## Conclusion

Among patients with NSTI, those with clostridial GG have a significantly higher mortality rate. In the initial stages, clinical differences are hardly detectable (see Table 5). CRP was significantly higher in clostridial GG compared to NF (*P* < 0.009), while LRINEC showed no significant difference. IL‐6 was highly elevated in both groups but without significant difference (*P* < 0.06). Immediate and repetitive surgical debridement is the key to successful therapy and needs to be performed as early as possible. In addition, full substitution with blood products, volume management, and broad and early antibiotic therapy are essential. Timely second‐look operations and reevaluation up to the decision of amputation are necessary.
